# Ionizing radiation enhances CAR T-cell infiltration and efficacy in solid tumors

**DOI:** 10.3389/fimmu.2026.1704419

**Published:** 2026-04-15

**Authors:** Lisa Zhan, Meredith A. Clark, Pauline Loos, Che-Min Lee, Laura E. R. Paulus, Rocky Shi, Prabhreet K. Sekhon, James M. Piret, Laura Evgin, Kevin L. Bennewith

**Affiliations:** 1 Basic and Translational Research, BC Cancer Research Institute, Vancouver, BC, Canada; 2 Interdisciplinary Oncology Program, University of British Columbia, Vancouver, BC, Canada; 3 Medical Genetics, University of British Columbia, Vancouver, BC, Canada; 4 Michael Smith Laboratories, University of British Columbia, Vancouver, BC, Canada; 5 Chemical and Biological Engineering, University of British Columbia, Vancouver, BC, Canada; 6 Pathology & Laboratory Medicine, University of British Columbia, Vancouver, BC, Canada

**Keywords:** CAR T cell therapy, ionizing radiation, melanoma, perfusion, solid tumors

## Abstract

Chimeric antigen receptor (CAR) T cells represent an exciting therapeutic strategy with improved survival outcomes for patients with hematological malignancies. However, the efficacy of CAR T-cell therapy in the treatment of solid tumors remains suboptimal due to therapeutic barriers associated with the solid tumor microenvironment. We investigated whether ionizing radiation could improve vascular perfusion and CAR T-cell delivery in an EGFRvIII-expressing B16F10 melanoma model. Tumors received radiation doses of 2–12 Gy, and perfusion was evaluated at multiple time points using immunofluorescence detection of intravenously administered fluorescent dyes. We found that a single 8-Gy dose of ionizing radiation produced the most significant increase in B16F10 tumor perfusion 4 h after irradiation. Consistently, the irradiation of tumors 4 h prior to a systemic administration of EGFRvIII-targeting CAR T cells led to higher intratumoral CAR T-cell accumulation than in non-irradiated tumors. This approach also resulted in a significantly delayed tumor growth and improved survival relative to radiation or CAR T cells alone. Interestingly, the CD28ζ EGFRvIII-CAR T-cell levels substantially increased in irradiated tumors over time relative to 4-1BBζ EGFRvIII-CAR T cells and produced greater tumor growth delays and survival improvements in comparison to 4-1BBζ EGFRvIII-CAR T cells administered at a 10-fold higher concentration. Taken together, these data highlight the importance of co-stimulatory domains in CAR T-cell function *in vivo* and demonstrate that irradiating tumors prior to systemic CAR T-cell infusion can increase CAR T-cell infiltration and efficacy in solid tumors.

## 1 Introduction

Over the past two decades, immunotherapy has emerged as a revolutionary treatment for patients with cancer in dire need of alternatives to surgery, chemotherapy, and radiotherapy (1). By utilizing a patient’s own immune system to eliminate cancer cells, immunotherapy has been proven to be indispensable in the fight against cancer. Chimeric antigen receptor (CAR) T-cell therapy is a form of immunotherapy where T cells are engineered to express a surface CAR molecule capable of recognizing tumor-associated antigens (2–4). Second-generation CAR molecules, which incorporate co-stimulatory domains such as 4-1BBζ (5) or CD28ζ (6), have produced CAR T-cell products with enhanced activation and persistence *in vivo*. These advances have led to the approval of seven U.S. Food and Drug Administration (FDA)-approved and six Health Canada-approved CAR T-cell therapies for hematological malignancies as of 2025 (7, 8). By targeting surface molecules such as CD19 or B-cell maturation antigen, CAR T cells have achieved significant success in the treatment of relapsed and refractory hematological malignancies such as B-cell acute lymphoblastic leukemia, large B-cell lymphoma, and multiple myeloma (7, 9–12).

Despite these successes, CAR T-cell therapy remains largely ineffective against solid tumors, which comprise over 70% of all cancer cases (13). A key challenge arises from the solid tumor microenvironment that can contain a poorly functional vasculature and limited blood flow that impedes CAR T-cell infiltration, antitumor activity, and persistence in tumors. Whereas CAR T cells targeting hematological malignancies can effectively access cancer cells within the bloodstream, lymphatics, or secondary lymphoid organs, CAR T cells aimed at solid tumors must penetrate the poorly perfused and dysfunctional vasculature of the tumor to reach their targets (14). To overcome these physical barriers and improve CAR T-cell therapy for solid tumors, current research has focused on combinatorial strategies targeting the tumor microenvironment, predicated on the understanding that complementary modulation of immune cell infiltration may synergistically enhance CAR T-cell efficacy (15).

Radiotherapy, a cornerstone of cancer care for over a century, remains widely used as both a stand-alone treatment and in combination with other therapeutic modalities (16). While traditionally aimed at inducing lethal DNA damage in cancer cells, radiotherapy can also impact the structure and function of the tumor vasculature within the radiation field (16). The vascular effects of radiation vary depending on several factors including the total dose, the fractionation schedule, and the tumor type, location, and stage. Notably, high radiation doses have been shown to decrease the tumor vascular volume (17, 18), whereas radiation doses lower than ~10 Gy can induce transient increases in tumor blood flow (19–21).

Building on this knowledge, we hypothesize that ionizing radiation (IR) targeted to the tumor site could act as a neoadjuvant therapy to enhance CAR T-cell delivery and efficacy by temporarily improving tumor vascular perfusion. To test this, we utilized immunocompetent C57Bl/6J mice bearing EGFRvIII-expressing B16F10 melanoma cells to evaluate the impact of various radiation doses and irradiation schedules on tumor perfusion. We then assessed how radiation affected the delivery of EGFRvIII-targeting CAR T cells manufactured with second-generation CAR constructs containing either a 4-1BBζ or a CD28ζ co-stimulatory domain and, ultimately, how radiation influenced the CAR T-cell efficacy in the model. Our findings support the use of targeted IR prior to CAR T-cell administration to enhance the infiltration and antitumor activity in solid tumors.

## 2 Materials and methods

### 2.1 Cell lines

Murine EGFRvIII-expressing B16F10 (EGFRvIII-B16F10), a gift from Dr. John Sampson (Duke University, Durham) (22), and wild-type B16F10 (ATCC) melanoma cell lines were cultured in Dulbecco’s modified Eagle’s medium (DMEM) (12100046; Gibco, Grand Island, NY, USA) supplemented with 10% fetal bovine serum (FBS) (12483-020, origin: Canada; Gibco). HEK 293T cells were cultured in DMEM with 10% FBS and 1× GlutaMAX™ (35050-061; Gibco). All cell lines tested negative for mycoplasma contamination. Cells were used within 10 passages from thaw for all experiments.

### 2.2 Mice

Female C57Bl/6J mice aged 9–13 weeks from the Jackson Laboratory were used for all experiments. The mice were housed in OptiMice cages on ventilated racks under specific pathogen-free conditions within a modified barrier facility in the Animal Resource Centre at the BC Cancer Research Institute. Mice were allowed to acclimatize for at least a week after delivery to the facility prior to any experiments. Animal experiments were performed in compliance with the requirements of the Canadian Council on Animal Care and the University of British Columbia Animal Care Committee under our approved animal protocols A21–0266 and A25-0214.

### 2.3 *In vivo* experiments

2.0 × 10^5^ EGFRvIII-B16F10 cells were implanted subcutaneously (SubQ) on the lower flank of anesthetized mice. Cells were suspended in a 100-μl mixture of serum-free DMEM and Matrigel^®^ basement membrane matrix (354234; Corning, Corning, NY, USA) at a 1:1 ratio. The mice were randomized into treatment groups 7 days post-implant. On the same day, tumor-bearing mice were lymphodepleted via an intraperitoneal (i.p.) injection of filter-sterilized 200 mg/kg cyclophosphamide (CTX) (13849; Cayman Chemical, Ann Arbor, MI, USA) dissolved in sterile 0.9% NaCl. Tumor irradiations were performed using a Precision X-ray machine (X-RAD320; 250 keV, ~3 Gy/min) 9 or 10 days post-implant as indicated. The mice were restrained in a lead jig that resulted in the radiation beam being targeted to the exposed tumor while shielding the rest of the mouse. CAR T cells were administered intravenously (i.v.) to anesthetized mice in 100 µl of serum-free RPMI-1640 media (10-040-CV; Corning).

Following CAR T-cell injections, the tumor volumes were measured every other day via caliper measurements (width^2^ × length × 0.5). Mice reached the humane endpoint when the tumor volumes reached approximately 5% of the mouse body weight. Tumor growth delay plots ended when at least two of the mice within a group reached the tumor volume limit. All mice were allowed to progress to the humane endpoint for the Kaplan–Meier survival analyses.

### 2.4 Tissue processing

Tumors resected for flow cytometry were finely chopped with scalpels and agitated at 37°C with 3 ml of serum-free DMEM and 2 ml of collagenase type I (4 mg/ml) (17100017; Gibco) and type II (2 mg/ml) (17101015; Gibco) for 20 min. We added 1 ml of DNase (3 mg/ml) (DN25-1G; Sigma-Aldrich, St. Louis, MO, USA) after the digestion was completed. The tumor samples were filtered through a 100-μm cell strainer to generate a single-cell suspension. Cells were counted using the Countess 3 Fl automated cell counter (ThermoFisher, Waltham, MA, USA).

### 2.5 Flow cytometry

Single-cell suspensions were washed with PBS and stained with Fixable Viability Dye eFluor 780 (65-0865-14; Invitrogen, Carlsbad, CA, USA) for 30 min on ice. The cells were then washed and suspended in an HFN solution consisting of Hank’s balanced salt solution (37150; STEMCELL Technologies, Vancouver, Canada) with 2% FBS + 0.05% NaN_3_ (S0209; Teknova, Hollister, CA, USA). The cells were blocked with anti-murine CD16/32 (93; 101302, BioLegend, San Diego, CA, USA) for 10 min on ice prior to antibody staining. The following monoclonal antibodies were used in a 15-min staining process on ice: CD4-SparkUV387 (GK1.5; 100492, BioLegend), CD8α-BUV563 (53-6.7; 748535, BD Biosciences, San Jose, CA, USA), CD3-BUV737 (145-2C11; 612771, BD Biosciences), CD45-APC (30-F11; 103112, BioLegend), CD44-BV605 (IM7; 103047, BioLegend), CD62L-AF700 (MEL-14; 104426, BioLegend), Thy1.1-FITC (OX-7; 202504, BioLegend), and EGFRvIII-PE (L8A4; Ab00184-23.0, Absolute Antibody, Redcar, Cleveland, UK). The cells were then fixed and permeabilized for 30 min using the FoxP3/Transcription Factor Buffer Staining Kit (00-5523-00; Invitrogen). All sample data were acquired on the FACSymphony™ A5 (BD Biosciences) cytometer and analyzed with FlowJo (FlowJo, LLC, Ashland, OR, USA).

### 2.6 Plasmids

The MSGV1 retroviral transfer plasmid contains the CAR cassette and a Thy1.1 marker gene, separated by an IRES element. The CAR cassette consists of a second-generation construct containing a single-chain antibody fragment (scFv) derived from the human monoclonal antibody 139 targeting EGFRvIII (23), a CD8 transmembrane domain, either a 4-1BBζ or a CD28ζ co-stimulatory domain, and a CD3ζ activation domain. The pCL-ECO packaging vector was a gift from Inder Verma (Verma Addgene plasmid #12371; https://www.addgene.org/12371/; RRID: Addgene_12371) (24) and is made up of a CMV–LXSN vector backbone and contains the *gag*/*pol*/*env* genes required for retroviral replication and transduction.

### 2.7 Murine CAR T-cell production

CAR T cells were generated using a previously published protocol (25). In brief, splenocytes were harvested from the spleens of naive female C57Bl/6J mice aged 9–13 weeks. T cells were activated with 10 ng/ml interleukin 7 (IL-7) (217-17-50UG; Peprotech, Cranbury, NJ, USA), 5 ng/ml IL-15 (210-15-10UG; Peprotech), and 30 ng/ml IL-21 (210-21-10UG; Peprotech) with 2.5 μg/ml concanavalin A (ConA) (J61221.MD; Invitrogen) in complete RPMI-1640 media supplemented with 10% heat-inactivated FBS (12484-028, origin: Canada; Gibco), 1 mM sodium pyruvate (S8636-100ML; Sigma-Aldrich), 100 U/ml penicillin/streptomycin (15140-122; Gibco), 50 μM 2-mercaptoethanol (60-24-2; Sigma-Aldrich), and 1× MEM non-essential amino acids (25-025-CI; Corning) for 2 days. IL-7 and IL-15 were added to the media for the complete duration of the CAR T-cell production period, with IL-21 and ConA present only for the initial 2 days. Retroviruses were produced using 3.5 × 10^6^ HEK 293T cells plated onto poly-d-lysine-coated (A38904-01; Gibco) plates. HEK 293T cells were transfected with a mixture of CAR plasmid (14.1 μg) and the packaging pCL Eco plasmid (9.9 μg) using Lipofectamine™ 2000 Transfection Reagent (11668019; Invitrogen). Transduction via spinoculation of activated T cells occurred 2 days after the initial T-cell activation in plates coated with the transduction enhancer recombinant HFN fragment (Retronectin) (T100B; Takara, Shiga, Japan). CAR T cells were split 1 day after the spinoculation and left to expand for another day afterward prior to any experiments.

The transduction efficiency was assessed 24 h after transduction and on the day of injection using flow cytometry to quantify the proportion of Thy1.1-expressing CAR T cells. The number of CAR T cells injected into mice was normalized to the Thy1.1^+^ cell count, with 10.0 × 10^6^ Thy1.1^+^ EGFRvIII.4-1BBζ CAR T cells or 1.0 × 10^6^ Thy1.1^+^ EGFRvIII.CD28ζ CAR T cells delivered per mouse.

### 2.8 *In vitro* co-culture assay

To validate the targeting capability of the CAR T cells, EGFRvIII.4-1BBζ CAR T cells, EGFRvIII.CD28ζ CAR T cells, or untransduced T cells (effector, E) were co-cultured with EGFRvIII-B16F10 cells (target, T) and wild-type B16F10 cells (non-target, NT). The same numbers of target to NT tumor cells were co-cultured with different numbers of effector cells. To differentiate the responses of target cells from those of NT cells, the EGFRvIII-B16F10 target cells were stained with Tag-it Violet™ (425101; BioLegend), while the NT wild-type B16F10 cells were stained with CellTracker™ Green CMFDA (C2925; Invitrogen). Wells with no CAR T cells added were used to normalize the T-to-NT ratio independent of T-cell killing. To assess the level of target cell-specific killing, different effector-to-target (E/T) ratios of CAR T cells were evaluated (1:1, 3:1, and 10:1). Following a 4-h co-culture, the cells were harvested, stained with Fixable Viability Dye eFluor 780 (65-0865-14; Invitrogen), and analyzed using flow cytometry as above. The numbers of live target cells were compared to the numbers of live NT cells across the different E/T ratios using the following equation:


$$(\frac{{\mathrm{Live Target}}_{1:1:1}÷\mathrm{Live Non}-{\mathrm{target}}_{1:1:1}}{{\mathrm{Live Target}}_{0:1:1}÷\mathrm{Live Non}-{\mathrm{target}}_{0:1:1}})\;\times 100\%\;$$

### 2.9 Immunofluorescence analyses

To assess vascular perfusion, the mice were i.v. injected with a fluorescent dye mixture containing 50 μl of 20 mg/ml Hoechst 33342 (H3570; Invitrogen) (26–29) and 3 mg/ml rhodamine-conjugated 2 MDa dextran (2 MDa) (D7139; Invitrogen) (26) 10 min prior to tumor resection. While Hoechst 33342 has been reported to transiently decrease tumor microcirculation in some xenograft models (30), this reagent has been used extensively to quantify the tumor blood flow and the radial diffusion gradients in a variety of solid tumors (29, 31, 32). As untreated and irradiated B16F10 tumors were injected with the same concentration of Hoechst 33342, any potential effects of the reagent on tumor blood flow would not interfere with the relative comparison of tumor perfusion. Excised tumors were placed in optimal cutting temperature (OCT) compound (4583; Sakura Finetek, Alphen aan den Rijn, the Netherlands) and frozen at −80°C. Tumor sections (10 μm thick) were cut using a cryostat (Leica, Wetzlar, Germany). In order to reduce sampling bias and to capture data from multiple tumor areas, three step sections 50 μm apart were collected for each tumor, with the quantitative data from these three sections combined into a single data point. Full face sections were first imaged for Hoechst 33342 and rhodamine–dextran fluorescence at ×10 magnification using a Zeiss AXIO Imager.Z2 microscope with automated scanning and image tiling. The tumor images used for analyses consisted of 30–50 tiled images. Tumor sections were then stained with 4′,6-diamidino-2-phenylindole (DAPI) prior to repeat imaging, where DAPI was used to indicate the nuclei and facilitate the demarcation of viable tumor areas in each section. The resulting Hoechst 33342, rhodamine–dextran, and DAPI images were then overlaid to generate an image file. The images were analyzed using ImageJ (FIJI).

**Figure 1:**
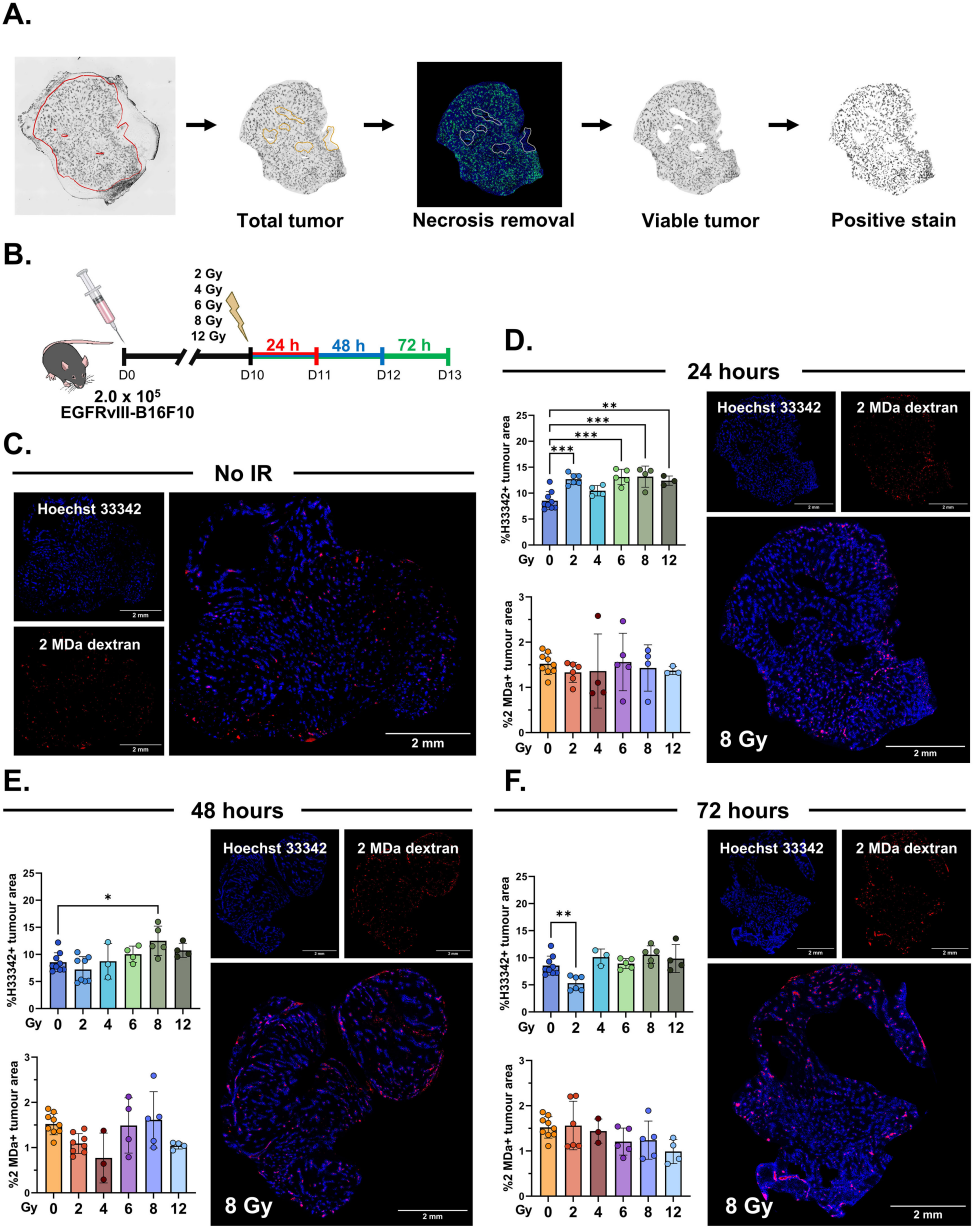
Changes in intravenously injected fluorescent perfusion dyes in tumors following ionizing radiation (IR) treatment. **(A)** Schematic showing the processing of tumor section scans to demarcate viable tumor regions and quantify fluorescent dye staining. **(B)** *In vivo* experiment schematic. **(C)** Representative immunofluorescent images of non-irradiated tumors. *Blue*, Hoechst 33342 (*H33342*); *red*, 2 MDa rhodamine-conjugated dextran (*2 MDa*). **(D)** Quantification of the tumor regions positive for H33342 (*top*) or 2 MDa (*bottom*) 24 h after various IR doses, with representative images of a tumor 24 h after 8 Gy. **(E)** Quantification of the tumor regions positive for H33342 (*top*) or 2 MDa (*bottom*) 48 h after various radiation doses, with representative images of a tumor 48 h after 8 Gy. **(F)** Quantification of the tumor regions positive for H33342 (*top*) or 2 MDa (*bottom*) 72 h after various radiation doses, with representative images of a tumor 72 h after 8 Gy. The illustrations in **(B)** are from the National Institute of Allergy and Infectious Diseases (NIAID) National Institute of Health (NIH) BioArt Source (bioart.niaid.nih.gov/bioart/506, bioart.niaid.nih.gov/bioart/281, bioart.niaid.nih.gov/bioart/298). Data points are individual tumors with *n* = 3–5 mice/group (IR-treated) and 12 mice/group (untreated), with the mean ± SEM indicated. *p ≤ 0.05; **p ≤ 0.01; ***p ≤ 0.001.


The total tumor area was first demarcated in each tiled image based on DAPI and Hoechst 33342 staining to exclude the surrounding Matrigel (if present) and OCT (**Figure 1A**). The resultant total tumor area was further analyzed to identify and exclude areas of necrosis, which were observed as regions of diffuse DAPI staining and reduced structural integrity of the section. The removal of necrotic areas from the total tumor image produced a mask of the viable tumor area that was used for downstream analyses. We then tested automated binarization threshold algorithms across a panel of untreated and treated tumors to identify the algorithm that produced binarized images that most accurately reflected the staining pattern observed in the non-binarized images. Li’s minimum cross-entropy thresholding method (33) was used to binarize all Hoechst images, and the triangle thresholding technique was used to binarize all rhodamine–dextran staining (34). Once a thresholding method was established for each stain, all treated and untreated tumors were processed using identical threshold settings for that stain. The thresholded binarized images were used to calculate the Hoechst- or rhodamine–dextran-positive areas in each section, which were then expressed as a percentage of the viable tumor area.

$$(\frac{\mathrm{Area of positive stain}}{\mathrm{Total viable tumor area}})\times 100\%=\%\;\mathrm{Positive tumor area}$$

### 2.10 Statistical analyses

All statistical analyses and graphs were generated using GraphPad Prism 10. The Shapiro–Wilk test was used to determine the normality of data distributions. A one-way ANOVA test was used to measure the mean between multiple groups with normally distributed data. When appropriate, a Brown–Forsythe test was used to determine significance with unequal variance between groups. Two-tailed Student’s *t*-tests were used to compare the means between two groups. For groups lacking a normal distribution, a non-parametric Kruskal–Wallis test was used to compare the means. Survival curves were constructed using the Kaplan–Meier method, and significance was assessed using the log-rank Mantel–Cox test. All data are reported as the mean ± standard error of the mean (SEM), unless otherwise specified.

## 3 Results

### 3.1 Radiation-induced increases in tumor perfusion

We first aimed to identify a radiation dose and a post-treatment time point that increase tumor perfusion to potentially enhance systemic CAR T-cell delivery to the tumor. Using immunofluorescence microscopy, we measured the tumor regions positive for i.v. injected Hoechst 33342 (vascular perfusion) and rhodamine-tagged 2 MDa dextran (2 MDa, large intravascular diameter) following various doses of external beam IR. We tested single irradiation doses ranging from 2 to 12 Gy and collected tumors at 24, 48, and 72 h post-irradiation (**Figure 1B**), comparing the perfusion levels to those of the non-irradiated controls (**Figure 1C**). The tumor masses at the time of harvest are indicated in **Supplementary Figures 1A–C**. We found that irradiated tumors had increased Hoechst 33342-positive tumor regions 24 h later (**Figure 1D**). The observed increases in Hoechst 33342-positive regions returned to the levels found in non-irradiated tumors by 48 h after radiation, with the exception of tumors treated with 8 Gy (**Figures 1E, F**). Radiation did not change the proportion of tumor area stained with intravascular 2 MDa dextran (**Figures 1D–F**).

**Figure 2:**
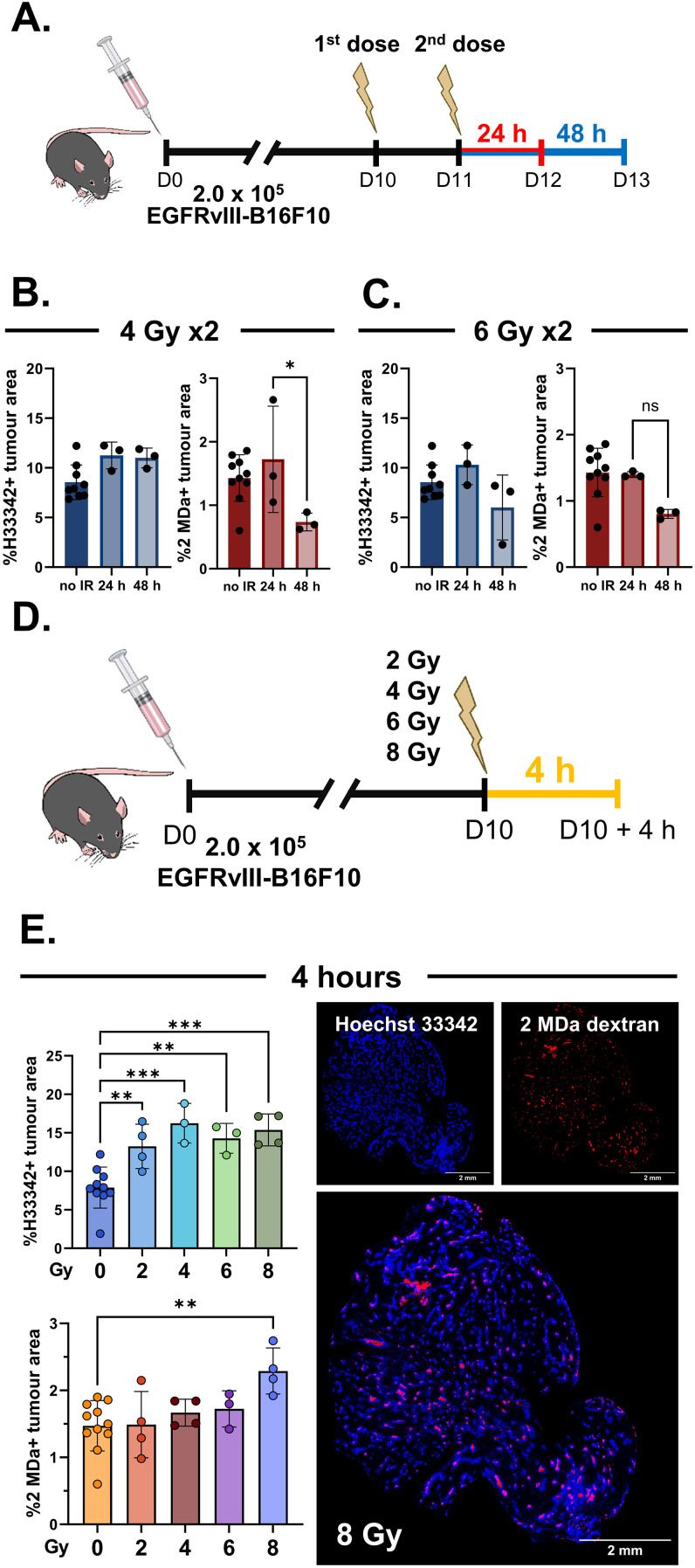
Fluorescent perfusion dye content in tumors 24–48 h following split-dose radiation or 4 h after a single radiation dose. **(A)** *In vivo* experiment schematic for the split-dose radiation regimen. **(B)** Quantification of the tumor regions positive for Hoechst 33342 (*H33342*) (*left*) or 2 MDa rhodamine-conjugated dextran (*2 MDa*) (*right*) following two doses of 4 Gy administered 24 h apart. **(C)** Quantification of the tumor regions positive for H33342 (*left*) or 2 MDa (*right*) following two doses of 6 Gy administered 24 h apart. **(D)** *In vivo* experiment schematic for tumors collected 4 h after a single radiation dose. **(E)** Quantification of the tumor regions positive for H33342 (*top*) or 2 MDa (*bottom*) 4 h after various single radiation doses, with representative images of a tumor 4 h after 8 Gy. The illustrations in **(A, D)** are from the National Institute of Allergy and Infectious Diseases (NIAID) National Institutes of Health (NIH) BioArt Source (bioart.niaid.nih.gov/bioart/506, bioart.niaid.nih.gov/bioart/281, bioart.niaid.nih.gov/bioart/298). Data points are individual tumors with *n* = 3–4 mice/group (ionizing radiation-treated) and 10–11 mice/group (untreated), with the mean ± SEM indicated. *p ≤ 0.05; **p ≤ 0.01; ***p ≤ 0.001.

We next examined whether splitting the total radiation dose into two fractions would alter tumor perfusion. We administered two fractions of 4 or 6 Gy 24 h apart (total doses of 8 and 12 Gy, respectively) and collected tumors 24 and 48 h after the second dose (**Figure 2A**). No increases in Hoechst 33342-positive or dextran-positive tumor regions were observed with either fractionated regimen (**Figures 2B, C**; **Supplementary Figure 1D**) at the assessed time points. These data indicate that while a single 8-Gy dose is sufficient to increase Hoechst 33342 delivery to tumors 24–48 h post-irradiation, splitting the dose into two fractions of 4 Gy does not recapitulate the findings during the same time window.

We postulated that radiation-induced changes in tumor perfusion may occur earlier than 24 h following an IR dose. Interestingly, we found that radiation doses ranging from 2 to 8 Gy significantly increased the proportion of Hoechst 33342-positive tumor regions 4 h post-irradiation (**Figures 2D, E**; **Supplementary Figure 1E**). We also observed increased intravascular 2 MDa dextran-positive regions 4 h after 8 Gy (**Figure 2E**), indicative of enlarged vascular structures in the tumor. Together, these findings demonstrate that IR can transiently enhance the intravenous delivery of fluorescent perfusion dyes to EGFRvIII-B16F10 tumors, with the most robust increase occurring 4 h after a dose of 8 Gy.

### 3.2 Generation of EGFRvIII-targeting CAR T cells

**Figure 3:**
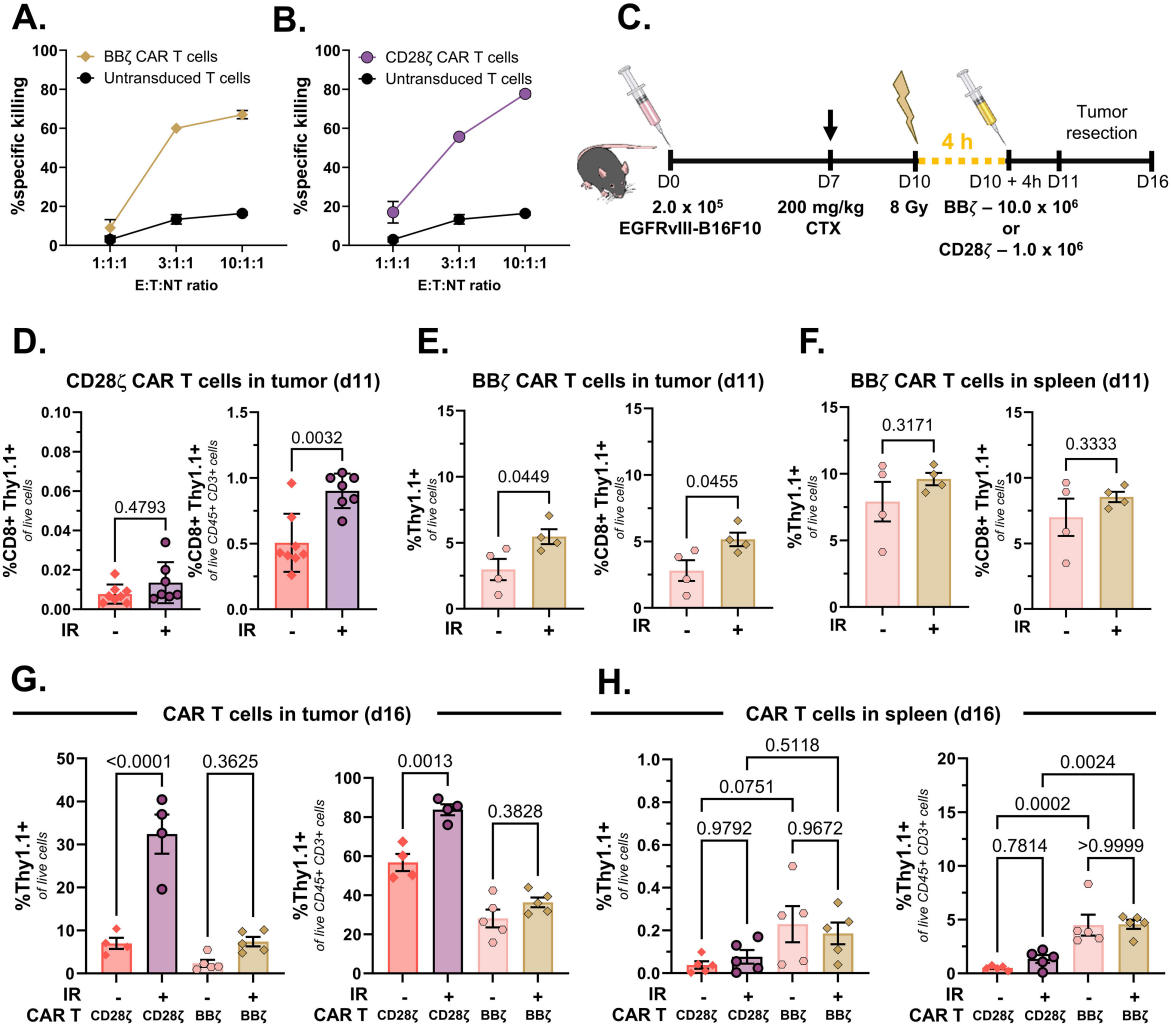
The chimeric antigen receptor (CAR) T-cell content is increased in tumors irradiated 4 h prior to systemic CAR T-cell injection. **(A)** Co-culture killing assay showing specific targeting of EGFRvIII.4-1BBζ CAR T cells (effectors, *E*) to EGFRvIII-B16F10 (target, *T*) cells at different E/T ratios. 1.0 × 10^4^ EGFRvIII-B16F10 cells and 1.0 × 10^4^ wild-type B16F10 (non-target, *NT*) cells (1:1) were incubated alongside different ratios of CAR T cells or non-transduced activated T cells. **(B)** Co-culture killing assay showing specific targeting of EGFRvIII.CD28ζ CAR T cells to EGFRvIII-B16F10 cells at different E/T ratios. **(C)** *In vivo* experiment schematic to assess CAR T-cell infiltration in irradiated or non-irradiated tumors. **(D)** Proportions of CD8^+^Thy1.1^+^ CAR T cells in tumors analyzed 24 h after injection of 1.0 × 10^6^ CD28ζ CAR T cells. **(E)** Proportions of total Thy1.1^+^ (*left*) and CD8+Thy1.1^+^ CAR T cells (*right*) in tumors analyzed 24 h after injection of 10.0 × 10^6^ 4-1BBζ CAR T cells. **(F)** Proportions of total Thy1.1^+^ (*left*) and CD8^+^Thy1.1^+^ (*right*) 4-1BBζ CAR T cells in spleens analyzed 24 h after injection. **(G)** Proportions of CD28ζ and 4-1BBζ CAR T cells in tumors analyzed 6 days after injection of 1.0 × 10^6^ CD28ζ or 10.0 × 10^6^ 4-1BBζ CAR T cells. **(H)** Proportions of CD28ζ and 4-1BBζ CAR T cells in spleens analyzed 6 days after injection. Data points are individual mice with a mean ± SEM of 4–8 mice/group, with *p*-values indicated. The illustrations from **(C)** are from the National Institute of Allergy and Infectious Diseases (NIAID) National Institutes of Health (NIH) BioArt Source (bioart.niaid.nih.gov/bioart/506, bioart.niaid.nih.gov/bioart/281, bioart.niaid.nih.gov/bioart/298).

Our next objective was to determine whether 8 Gy of IR would increase the delivery of CAR T cells injected systemically 4 h after tumor irradiation. We first generated murine CAR T cells designed to target the surface molecule EGFRvIII, incorporating either a 4-1BBζ (BBζ) or a CD28ζ co-stimulatory domain. Regardless of the co-stimulatory domain, the resultant CAR T cells were typically ~80% CD8^+^ and 10%–15% CD4^+^, with 70%–85% transduction efficiency based on Thy1.1 expression and similar phenotypes as measured by the expression of the activation markers CD44 and CD62L (**Supplementary Figures 2A, B**). To assess CAR T-cell-specific targeting against EGFRvIII-B16F10 cells *in vitro*, we combined EGFRvIII-B16F10 target cells and wild-type B16F10 NT cells at a 1:1 ratio and introduced either CAR T cells or untransduced activated T cells (effector; E) at three different E/T/NT ratios (1:1:1, 3:1:1, and 10:1:1). The presence of surface EGFRvIII exclusively on EGFRvIII-B16F10 tumor cells and not on wild-type B16F10 cells was confirmed via flow cytometry (**Supplementary Figures 2C, D**). EGFRvIII-targeting CAR T cells with either the 4-1BBζ or the CD28ζ co-stimulatory domain showed specific killing of EGFRvIII-B16F10 cells following a 4-h co-culture (**Figures 3A, B**). CAR T cells containing either co-stimulatory domain showed similar levels of target-specific killing *in vitro*. The EGFRvIII-targeting CAR T cells generated using these constructs were subsequently used for *in vivo* experiments.

### 3.3 Increased CAR T-cell infiltration in previously irradiated tumors

Lymphodepletion with fludarabine and/or CTX is often used to enhance CAR T-cell expansion and persistence *in vivo*. Indeed, tumor-bearing mice injected with CTX had significantly reduced immune cells (including NK cells, B cells, and T cells) in tumors 3 days later (**Supplementary Figure 3**). EGFRvIII-B16F10-bearing mice were therefore lymphodepleted 3 days prior to irradiation and systemic CAR T-cell injection, with the intratumoral CAR T-cell content quantified 24 h later (**Figure 3C**). We i.v. injected 1.0 × 10^6^ Thy1.1^+^ EGFRvIII-targeting CD28ζ CAR T cells and found nearly twofold higher levels of CAR T cells in tumors irradiated 4 h prior to CAR T-cell injection (**Figure 3D**). In order to increase the detection sensitivity of our analysis, we next i.v. injected 10.0 × 10^6^ Thy1.1^+^ EGFRvIII-targeting 4-1BBζ CAR T cells (**Supplementary Figure 4A**) and similarly found nearly twofold more CAR T cells in tumors irradiated 4 h prior to CAR T-cell injection (**Figure 3E**). Notably, the Thy1.1^+^ 4-1BBζ CAR T-cell levels in the spleens were similar between mice with irradiated or non-irradiated tumors, indicating that the higher intratumoral CAR T-cell presence was not due to differences in the overall engraftment (**Figure 3F**).

While tumor irradiation increased the infiltration of CD28ζ and 4-1BBζ CAR T cells evaluated 24 h after CAR T-cell injection, we were interested in whether the intratumoral CAR T-cell levels would remain elevated over a longer period. We again injected 1.0 × 10^6^ CD28ζ or 10.0 × 10^6^ 4-1BBζ EGFRvIII-targeting CAR T cells 4 h after tumor irradiation with 8 Gy, analyzing the intratumoral CAR T-cell content 6 days later. The tumor volumes at the time of CAR T-cell injection and the weights at the time of tumor harvest are shown in **Supplementary Figure 4B**. We found that the proportions of live CD28ζ or 4-1BBζ CAR T cells substantially increased in tumors compared with those at 1 day after CAR T-cell injection. The CD28ζ CAR T-cell levels expanded dramatically in both non-irradiated and irradiated tumors (**Figure 3G**), with significantly higher levels of CAR T cells in irradiated tumors. Interestingly, CD28ζ CAR T cells made up a higher proportion of intratumoral T cells compared with 4-1BBζ CAR T cells, despite injecting 10-fold higher numbers of 4-1BBζ CAR T cells 6 days earlier. We again did not observe differences in the CAR T-cell content in the spleens between mice with or without tumor irradiation (**Figure 3H**), and the lower frequency of CD28ζ CAR T cells relative to 4-1BBζ CAR T cells in the spleens suggests preferential tumor-specific expansion of CD28ζ CAR T cells, particularly in irradiated tumors.

### 3.4 Delayed tumor growth and improved survival following 8-Gy irradiation 4 h before CAR T-cell delivery

**Figure 4:**
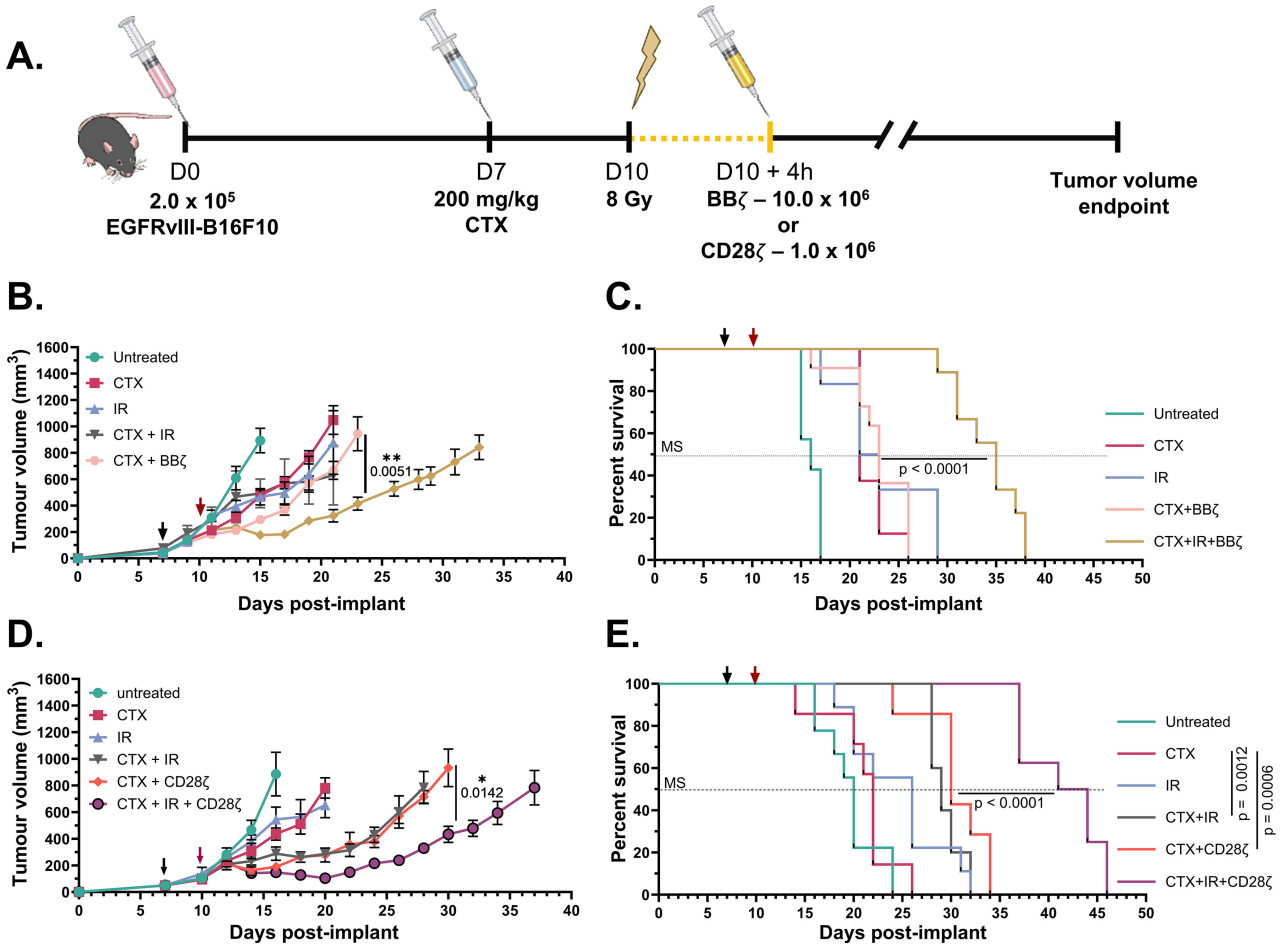
Injecting chimeric antigen receptor (CAR) T cells 4 h after tumor irradiation increases tumor growth delay and improves survival. **(A)** *In vivo* experiment schematic determining tumor growth delay and survival time after treatment. **(B)** Tumor growth delay curves for EGFRvIII.B16F10 tumor-bearing mice that were untreated, lymphodepleted with cyclophosphamide (CTX), treated with 8 Gy ionizing radiation (IR), and/or intravenously (i.v.) injected with 10.0 × 10^6^ Thy1.1^+^ 4-1BBζ CAR T cells 4 h after IR. Data are the mean ± SEM of 5–11 mice/group. **(C)** Kaplan–Meier curves showing the survival of tumor-bearing mice in **(B)**. **(D)** Tumor growth delay curves for EGFRvIII.B16F10 tumor-bearing mice that were untreated, lymphodepleted with CTX, treated with 8 Gy IR, and/or i.v. injected with 1.0 × 10^6^ Thy1.1^+^ CD28ζ CAR T cells 4 h after IR. Data are the mean ± SEM of 5–9 mice/group. **(E)** Kaplan–Meier curves showing the survival of tumor-bearing mice in **(D)**. *MS* denotes line to visualize the 50% median survival for each cohort. *Black arrow* indicates CTX injection, and *red arrow* indicates irradiation and/or CAR T-cell injection. The illustrations in **(A)** are from the National Institute of Allergy and Infectious Diseases (NIAID) National Institutes of Health (NIH) BioArt Source (bioart.niaid.nih.gov/bioart/506, bioart.niaid.nih.gov/bioart/281, bioart.niaid.nih.gov/bioart/298). *p≤0.05, **p≤0.01, ***p≤0.001.

We then examined whether irradiating tumors with 8 Gy 4 h prior to systemic CAR T-cell injection, as well as the resultant increase in the intratumoral CAR T-cell content, would lead to superior tumor control and prolonged overall survival. As above, we lymphodepleted mice with CTX 7 days after EGFRvIII-B16F10 tumor implant, followed 3 days later by tumor irradiation and/or i.v. injection of CAR T cells (**Figure 4A**). The cohort of untreated mice reached the tumor volume limits 15 days after tumor implant (**Figure 4B**), with a median survival on day 16 (**Figure 4C**). It should be noted that the average tumor growth delay plots were continued until at least two of the mice within a group reached the tumor volume limit, while all mice in a group were allowed to progress to the humane endpoint for the Kaplan–Meier survival analyses. Mice treated with a monotherapy of either CTX or IR had delayed tumor growth and an extended median survival time of 21–23 days. Mice treated with a combination of CTX and IR (CTX+IR) had similar tumor growth trends to mice treated with a combination of CTX and 10.0 × 10^6^ 4-1BBζ CAR T cells (CTX+BBζ). While the survival analysis could not be completed for the CTX+IR cohort due to a high number of non-tumor volume-related euthanasia, mice in the CTX+BBζ cohort reached the tumor volume limits and median survival 23 days post-implant. We found that lymphodepleted mice that had been irradiated 4 h prior to delivery of 10.0 × 10^6^ 4-1BBζ CAR T cells (CTX+IR+BBζ) had significantly delayed tumor growth to 33 days post-implant and a median survival time of 35 days (**Figures 4B, C**; **Supplementary Figure 5A**).

We performed a similar experiment using i.v. injection of 1.0 × 10^6^ CD28ζ EGFRvIII-targeting CAR T cells 4 h after tumor irradiation (**Figure 4A**). In line with previous experiments, untreated mice reached the tumor volume limits 16 days post-implant, with a median survival of 20 days post-implant, while mice treated with CTX alone or IR alone reached the tumor volume limits by 20 days post-implant, with median survival times of 22–26 days (**Figures 4D, E**). Mice lymphodepleted with CTX 3 days prior to IR (CTX+IR) or delivery of 1.0 × 10^6^ CD28ζ CAR T cells (CTX+CD28ζ) showed similar tumor growth delays, with both groups reaching the tumor volume limits and median survival by 29–30 days post-implant. Consistent with the data in **Figures 4B, C**, CTX-lymphodepleted mice that received 8 Gy 4 h prior to CD28ζ CAR T-cell injection (CTX+IR+CD28ζ) exhibited significantly delayed tumor growth to 37 days post-implant and a median survival time of 44 days (**Figures 4D, E**; **Supplementary Figure 5B**). It is worth noting that CD28ζ CAR T cells induced greater tumor growth delays and longer median survival times than 4-1BBζ CAR T cells injected at 10-fold higher concentrations, which is consistent with the increased numbers of CD28ζ CAR T cells relative to 4-1BBζ CAR T cells in tumors 6 days after injection (**Figure 3G**).

Lastly, we compared the intratumoral CAR T-cell content and efficacy when 1.0 × 10^6^ CD28ζ CAR T cells were injected 24 h (instead of 4 h) after tumor irradiation with 8 Gy. We found that injection of CAR T cells 24 h after tumor irradiation produced variable numbers of intratumoral CAR T cells that were not significantly different from those in mice injected with CAR T cells 4 h after tumor irradiation (**Supplementary Figure 5C**). We also found that mice injected with 1.0 × 10^6^ CD28ζ CAR T cells 24 h after tumor irradiation reached the tumor volume limits by day 33 and therefore had delayed tumor growth relative to mice treated with CTX+IR or CTX+CD28ζ (**Supplementary Figure 5D**). The treatment-induced growth delay was slightly shorter than that in mice injected with 1.0 × 10^6^ CD28ζ CAR T cells 4 h after tumor irradiation, where the tumor volume limits were reached by day 35. Therefore, injection of CAR T cells 4 or 24 h after tumor irradiation results in an enhanced intratumoral CAR T-cell content and an improved therapeutic efficacy, consistent with radiation-induced increases in tumor perfusion over this time frame.

Collectively, our data demonstrate that IR can increase perfusion and CAR T-cell infiltration into murine melanoma tumors and that CAR T cells constructed with a CD28ζ co-stimulatory domain are more abundant in tumors and have better efficacy than CAR T cells constructed with a 4-1BBζ co-stimulatory domain administered at 10-fold higher concentrations.

## 4 Discussion

In this study, we identified an IR dose and a time frame that enhance the vascular perfusion of EGFRvIII-B16F10 melanoma tumors. A single 8-Gy dose increased tumor perfusion 4 h after irradiation, as measured by the increased delivery of i.v. injected Hoechst 33342 and the increased intravascular 2 MDa dextran staining. This effect was observed to be transient, with tumor perfusion returning to baseline levels within 72 h, thereby identifying a therapeutic window for maximizing the systemic delivery of immunotherapies to the tumor. We also found that irradiating tumors 4 h prior to CAR T-cell injection resulted in improved intratumoral CAR T-cell levels and increased efficacy compared with that in mice treated with radiation or CAR T-cell injection alone. We compared the EGFRvIII-targeting CAR T cells generated with a 4-1BBζ or a CD28ζ co-stimulatory domain and found that, despite similar killing capacity *in vitro* (**Figures 3A, B**), the CD28ζ CAR T cells showed an increased intratumoral accumulation over time and an improved efficacy compared with the 4-1BBζ CAR T cells delivered at a 10-fold higher concentration (**Figures 4B–E**). Our data highlight the therapeutic importance of CAR T-cell co-stimulatory domains and indicate that IR can be used to increase the CAR T-cell infiltration and efficacy in solid tumors.

Despite the clinical successes observed with CAR T cells against hematological malignancies, CAR T cells have not shown consistent efficacy against solid tumors, where the solid tumor microenvironment can severely limit their infiltration and activity. Several strategies to improve CAR T-cell function in tumors have been assessed, including the use of oncolytic viruses to prime tumor cells (35, 36), restoring CAR T-cell function using anti-PD/PD-L1 immune checkpoint blockade (37, 38), or the degradation of extracellular matrix components within tumors to increase CAR T-cell infiltration (39). However, none of these strategies address the presence of an aberrant tumor vasculature and the resulting poor tumor blood flow that can severely limit CAR T-cell trafficking in solid tumors (40, 41). While certain treatments such as vasodilators and carbogen (5% CO_2_ and 95% O_2_) inhalation can increase blood flow in patients, research has shown variable effects in the context of increasing blood flow, particularly in solid tumors. The response to vasodilators is affected by the structure of the tumor vasculature, which is generally known to be immature and often in a state of near maximal dilation (42). The systemic vasodilatory response to vasodilators can also lead to a phenomenon known as “the steal effect,” where the increased blood flow to other tissues in the body causes decreased blood flow to solid tumors (43–45). While carbogen inhalation increases the oxygen-carrying capacity of the blood, the effect of carbogen on tumor perfusion is highly variable (46). Strategies such as monoclonal antibodies targeting VEGF-A (41, 47) or kinases critical in the coordination of blood vessel development (48, 49) have also been studied; however, they lack the availability, simplicity, and the relatively low cost of alternative readily available therapies.

IR can influence the functionality of tumor blood vessels (16), with radiation doses below ~10 Gy capable of inducing transient increases in tumor blood flow (19–21). We used Hoechst 33342 and intravascular rhodamine–dextran staining to indicate a snapshot of tumor blood flow during a 10-min window between dye injection and tumor harvest. It should therefore be noted that the distribution of these fluorescent reagents is not able to discriminate between the radiation-induced changes in functional tumor perfusion, the vascular permeability, or the endothelial cell–cell junction integrity. However, regardless of the mechanism, our imaging data indicate that the tumor blood flow increased 4 h after tumor irradiation, providing a potential window of opportunity to improve the delivery of systemically injected CAR T cells. IR is an attractive partner for CAR T-cell therapy as radiation can be precisely delivered to the tumor mass to minimize normal tissue cytotoxicity and, as a neoadjuvant therapy, will not adversely impact the survival of the subsequently administered CAR T cells (unlike systemic chemotherapies). To date, several studies have reported how radiation can be leveraged to improve CAR T-cell therapy. Radiotherapy can increase antigen presentation, thereby mitigating antigen escape, as shown in pancreatic (50), glioblastoma (51), and prostate tumor models (52). It has also been shown to enhance CAR T-cell efficacy in a lymphoma model by increasing antigen cross-presentation and cGAS/STING activation (53). In triple-negative breast cancer models, radiotherapy upregulated Icam-1 expression on tumor cells, consequently increasing the activation of NF-κB signaling (49). A study focusing on the effect of radiotherapy on extracellular matrix stiffness and plasticity has been published using gastric carcinoma and colorectal tumor models (54). Irradiating the thoracic cavity of immunocompromised mice with 4 Gy 3 days prior to CAR T-cell infusion resulted in improved efficacy in mesothelioma and lung cancer models (55). Here, we used IR to increase the perfusion of B16F10 tumors with the goal of improving CAR T-cell delivery and found that 8 Gy administered 4 h prior to systemic CAR T-cell injection did indeed increase the intratumoral CAR T-cell content assessed 1 or 6 days later. While our study was not designed to discriminate alternative effects of IR on CAR T-cell efficacy in this model, we postulate that the combination of radiation-induced increases in tumor perfusion with other known mechanisms of radiation-associated improvements in CAR T-cell efficacy makes IR a particularly attractive neoadjuvant therapy to improve CAR T-cell function in solid tumors.

Our study also highlights the influence of the co-stimulatory domains on CAR molecules on the ability of CAR T cells to accumulate in solid tumors after irradiation and reduce tumor growth. Four out of the six approved CAR T-cell therapies use a 4-1BBζ co-stimulatory domain, which is associated with greater persistence and reduced severity in cytokine release syndrome and neurotoxicity (56–58). However, emerging evidence suggests that CD28ζ CAR T cells may be more effective against solid tumors (59, 60). Indeed, in our melanoma model, CD28ζ CAR T cells showed significantly higher intratumoral accumulation than 4-1BBζ CAR T cells 6 days after systemic injection, despite being administered at 1/10 of the concentration. CD28ζ CAR T cells also induced greater tumor growth delays and increased survival of tumor-bearing mice compared with mice given 10-fold higher levels of 4-1BBζ CAR T cells. Reducing the number of injected CAR T cells can decrease the risk of CAR T-cell-associated morbidities, including cytokine release syndrome, and our data indicate how the choice of co-stimulatory domain can be used to optimize CAR T-cell delivery and efficacy in solid tumors.

One of the limitations of this study is our choice of CAR target. EGFRvIII is a candidate marker for CAR T cells targeting glioblastoma cells, but is not inherently expressed by melanoma cells. Furthermore, the majority of the EGFRvIII-B16F10 tumor cells in our model expressed high levels of EGFRvIII, while endogenous target molecule expression in patient tumors is typically more heterogeneous. Nevertheless, this model provides proof-of-concept data to support the further testing of CAR T-cell administration within hours of solid tumor irradiation. The effects of radiation will also have to be examined in other tumor models to ascertain how radiation-induced changes to the tumor vasculature may differ between various tumor models. Further research is needed to fully explore the different ways that IR can be most effectively combined with CAR T-cell therapy to produce the greatest impact on the treatment of solid tumors.

Taken together, our data indicate that treatment of solid tumors with IR prior to systemic CAR T-cell injection may be a viable strategy to increase the intratumoral CAR T-cell levels and improve therapeutic efficacy. Our approach proved efficacious in slowing tumor growth and prolonging survival using two different second-generation CAR molecules, underpinning the feasibility of this treatment strategy with different CAR T cells. These results lay the groundwork for future studies to examine IR as a modulator of the solid tumor microenvironment, with the ultimate goal of improving CAR T-cell-based therapies for solid malignancies.

## Data Availability

The raw data supporting the conclusions of this article will be made available by the authors, without undue reservation.

## References

[1] Dobosz P Dzieciątkowski T . The intriguing history of cancer immunotherapy. Front Immunol. (2019) 10:2965. doi: 10.3389/fimmu.2019.02965. PMID: 31921205 PMC6928196

[2] Chen YJ Abila B Mostafa Kamel Y . CAR-T: what is next? Cancers (Basel). (2023) 15:663. doi: 10.3390/cancers15030663. PMID: 36765623 PMC9913679

[3] Kalos M Levine BL Porter DL Katz S Grupp SA Bagg A . T cells with chimeric antigen receptors have potent antitumor effects and can establish memory in patients with advanced leukemia. Sci Transl Med. (2011) 3:95ra73–3. doi: 10.1126/scitranslmed.3002842. PMID: 21832238 PMC3393096

[4] Schuster SJ Svoboda J Chong EA Nasta SD Mato AR Anak Ö . Chimeric antigen receptor T cells in refractory B-cell lymphomas. N Engl J Med. (2017) 377:2545–54. doi: 10.1056/NEJMoa1708566. PMID: 29226764 PMC5788566

[5] Imai C Mihara K Andreansky M Nicholson IC Pui CH Geiger TL . Chimeric receptors with 4-1BB signaling capacity provoke potent cytotoxicity against acute lymphoblastic leukemia. Leukemia. (2004) 18:676–84. doi: 10.1038/sj.leu.2403302. PMID: 14961035

[6] Maher J Brentjens RJ Gunset G Rivière I Sadelain M . Human T-lymphocyte cytotoxicity and proliferation directed by a single chimeric TCRζ /CD28 receptor. Nat Biotechnol. (2002) 20:70–5. doi: 10.1038/nbt0102-70. PMID: 11753365

[7] O’Leary MC Lu X Huang Y Lin X Mahmood I Przepiorka D . FDA approval summary: Tisagenlecleucel for treatment of patients with relapsed or refractory B-cell precursor acute lymphoblastic leukemia. Clin Cancer Res. (2019) 25:1142–6. doi: 10.1158/1078-0432.CCR-18-2035. PMID: 30309857

[8] Summary Safety Review - Breyanzi (lisocabtagene maraleucel), Carvykti (ciltacabtagene autoleucel), Kymriah (tisagenlecleucel), Tecartus (brexucabtagene autoleucel) and Yescarta (axicabtagene ciloleucel) - Chimeric Antigen Receptor T-cell (CAR-T) Therapies - Assessing the Potential Risk of Secondary T-cell Malignancy. Available online at: https://dhpp.hpfb-dgpsa.ca/review-documents/resource/SSR1733492722458 (Accessed June 5, 2025).

[9] Timmers M Roex G Wang Y Campillo-Davo D Van Tendeloo VFI Chu Y . Chimeric antigen receptor-modified T cell therapy in multiple myeloma: beyond B cell maturation antigen. Front Immunol. (2019) 10:1613. doi: 10.3389/fimmu.2019.01613. PMID: 31379824 PMC6646459

[10] Turtle CJ Hanafi LA Berger C Gooley TA Cherian S Hudecek M . CD19 CAR–T cells of defined CD4^+^:CD8^+^ composition in adult B cell ALL patients. J Clin Invest. (2016) 126:2123–38. doi: 10.1172/JCI85309. PMID: 27111235 PMC4887159

[11] Maude SL Laetsch TW Buechner J Rives S Boyer M Bittencourt H . Tisagenlecleucel in children and young adults with B-cell lymphoblastic leukemia. N Engl J Med. (2018) 378:439–48. doi: 10.1056/NEJMoa1709866. PMID: 29385370 PMC5996391

[12] Neelapu SS Locke FL Bartlett NL Lekakis LJ Miklos DB Jacobson CA . Axicabtagene ciloleucel CAR T-cell therapy in refractory large B-cell lymphoma. N Engl J Med. (2017) 377:2531–44. doi: 10.1056/NEJMoa1707447. PMID: 29226797 PMC5882485

[13] Hou B Tang Y Li W Zeng Q Chang D . Efficiency of CAR-T therapy for treatment of solid tumor in clinical trials: a meta-analysis. Dis Markers. (2019) 2019:3425291. doi: 10.1155/2019/3425291. PMID: 30886654 PMC6388318

[14] Anderson NM Simon MC . Tumor microenvironment. Curr Biol. (2020) 30:R921–5. doi: 10.1016/j.cub.2020.06.081. PMID: 32810447 PMC8194051

[15] Al-Haideri M Tondok SB Safa SH Maleki AH Rostami S Jalil AT . CAR-T cell combination therapy: the next revolution in cancer treatment. Cancer Cell Int. (2022) 22:365. doi: 10.1186/s12935-022-02778-6. PMID: 36419058 PMC9685957

[16] Köry J Narain V Stolz BJ Kaeppler J Markelc B Muschel RJ . Enhanced perfusion following exposure to radiotherapy: a theoretical investigation. PloS Comput Biol. (2024) 20:e1011252. doi: 10.1371/journal.pcbi.1011252. PMID: 38363799 PMC10903964

[17] Song CW Payne JT Levitt SH . Vascularity and blood flow in X-irradiated Walker carcinoma 256 of rats. Radiology. (1972) 104:693–7. doi: 10.1148/104.3.693. PMID: 5051488

[18] Solesvik OV Rofstad EK Brustad T . Vascular changes in a human Malignant melanoma xenograft following single-dose irradiation. Radiat Res. (1984) 98:115–28. doi: 10.2307/3576056. PMID: 6718687

[19] Wong HH Song CW Levitt SH . Early changes in the functional vasculature of Walker carcinoma 256 following irradiation. Radiology. (1973) 108:429–34. doi: 10.1148/108.2.429. PMID: 4719050

[20] Dewhirst MW Oliver R Tso CY Gustafson C Secomb T Gross JF . Heterogeneity in tumor microvascular response to radiation. Int J Radiat Oncol Biol Phys. (1990) 18:559–68. doi: 10.1016/0360-3016(90)90061-N. PMID: 2318688

[21] Sonveaux P Dessy C Brouet A Jordan BF Grégoire V Gallez B . Modulation of the tumor vasculature functionality by ionizing radiation accounts for tumor radiosensitization and promotes gene delivery. FASEB J. (2002) 16:1979–81. doi: 10.1096/fj.02-0487fje. PMID: 12397083

[22] Sampson JH Crotty LE Lee S Archer GE Ashley DM Wikstrand CJ . Unarmed, tumor-specific monoclonal antibody effectively treats brain tumors. Proc Natl Acad Sci. (2000) 97:7503–8. doi: 10.1073/pnas.130166597. PMID: 10852962 PMC16575

[23] Sampson JH Choi BD Sanchez-Perez L Suryadevara CM Snyder DJ Flores CT . EGFRvIII mCAR-modified T-cell therapy cures mice with established intracerebral glioma and generates host immunity against tumor-antigen loss. Clin Cancer Res. (2014) 20:972–84. doi: 10.1158/1078-0432.CCR-13-0709. PMID: 24352643 PMC3943170

[24] Naviaux RK Costanzi E Haas M Verma IM . The pCL vector system: rapid production of helper-free, high-titer, recombinant retroviruses. J Virol. (1996) 70:5701–5. doi: 10.1128/jvi.70.8.5701-5705.1996. PMID: 8764092 PMC190538

[25] Loos P Short L Savage G Evgin L . Expansion and retroviral transduction of primary murine T cells for CAR T-cell therapy. Methods Mol Biol. (2024) 2748:41–53. doi: 10.1007/978-1-0716-3593-3_4. PMID: 38070106

[26] Potiron VA Abderrahmani R Clément-Colmou K Marionneau-Lambot S Oullier T Paris F . Improved functionality of the vasculature during conventionally fractionated radiation therapy of prostate cancer. PloS One. (2013) 8:e84076. doi: 10.1371/journal.pone.0084076. PMID: 24391887 PMC3877206

[27] Smith KA Hill SA Begg AC Denekamp J . Validation of the fluorescent dye Hoechst 33342 as a vascular space marker in tumours. Br J Cancer. (1988) 57:247–53. doi: 10.1038/bjc.1988.54. PMID: 3355762 PMC2246513

[28] Durand RE Olive PL . Cytotoxicity, mutagenicity and DNA damage by Hoechst 33342. J Histochem Cytochem. (1982) 30:111–6. doi: 10.1177/30.2.7061816. PMID: 7061816

[29] Wadsworth BJ Lee CM Bennewith KL . Transiently hypoxic tumour cell turnover and radiation sensitivity in human tumour xenografts. Br J Cancer. (2022) 126:1616–26. doi: 10.1038/s41416-021-01691-5. PMID: 35031765 PMC9130130

[30] Trotter MJ Olive PL Chaplin DJ . Effect of vascular marker Hoechst 33342 on tumour perfusion and cardiovascular function in the mouse. Br J Cancer. (1990) 62:903–8. doi: 10.1038/bjc.1990.406. PMID: 2257217 PMC1971547

[31] Wadsworth BJ Cederberg RA Lee CM Firmino NS Franks SE Pan J . Angiotensin II type 1 receptor blocker telmisartan inhibits the development of transient hypoxia and improves tumour response to radiation. Cancer Lett. (2020) 493:31–40. doi: 10.1016/j.canlet.2020.07.015. PMID: 32763272

[32] Wadsworth BJ Pan J Dude I Colpo N Bosiljcic M Lin KS . 2-18F-fluoroethanol is a PET reporter of solid tumor perfusion. J Nucl Med. (2017) 58:815–20. doi: 10.2967/jnumed.116.183624. PMID: 28126891

[33] Li CH Lee CK . Minimum cross entropy thresholding. Pattern Recognit. (1993) 26:617–25. doi: 10.1016/0031-3203(93)90115-D

[34] Zack GW Rogers WE Latt SA . Automatic measurement of sister chromatid exchange frequency. J Histochem Cytochem. (1977) 25:741–53. doi: 10.1177/25.7.70454. PMID: 70454

[35] Evgin L Kottke T Tonne J Thompson J Huff AL van Vloten J . Oncolytic virus–mediated expansion of dual-specific CAR T cells improves efficacy against solid tumors in mice. Sci Transl Med. (2022) 14:eabn2231. doi: 10.1126/scitranslmed.abn2231. PMID: 35417192 PMC9297825

[36] Moon EK Wang LCS Bekdache K Lynn RC Lo A Thorne SH . Intra-tumoral delivery of CXCL11 via a vaccinia virus, but not by modified T cells, enhances the efficacy of adoptive T cell therapy and vaccines. Oncoimmunology. (2018) 7:e1395997. doi: 10.1080/2162402X.2017.1395997. PMID: 29399394 PMC5790399

[37] Song Y Liu Q Zuo T Wei G Jiao S . Combined antitumor effects of anti-EGFR variant III CAR-T cell therapy and PD-1 checkpoint blockade on glioblastoma in mouse model. Cell Immunol. (2020) 352:104112. doi: 10.1016/j.cellimm.2020.104112. PMID: 32305131

[38] Cherkassky L Morello A Villena-Vargas J Feng Y Dimitrov DS Jones DR . Human CAR T cells with cell-intrinsic PD-1 checkpoint blockade resist tumor-mediated inhibition. J Clin Invest. (2016) 126:3130–44. doi: 10.1172/JCI83092. PMID: 27454297 PMC4966328

[39] Caruana I Savoldo B Hoyos V Weber G Liu H Kim ES . Heparanase promotes tumor infiltration and antitumor activity of CAR-redirected T-lymphocytes. Nat Med. (2015) 21:524–9. doi: 10.1038/nm.3833. PMID: 25849134 PMC4425589

[40] Ribatti D Pezzella F . Overview on the different patterns of tumor vascularization. Cells. (2021) 10:639. doi: 10.3390/cells10030639. PMID: 33805699 PMC8000806

[41] Ribatti D . Aberrant tumor vasculature. Facts and pitfalls. Front Pharmacol. (2024) 15:1384721. doi: 10.3389/fphar.2024.1384721. PMID: 38576482 PMC10991687

[42] Siemann DW . The unique characteristics of tumor vasculature and preclinical evidence for its selective disruption by tumor-vascular disrupting agents. Cancer Treat Rev. (2011) 37:63–74. doi: 10.1016/j.ctrv.2010.05.001. PMID: 20570444 PMC2958232

[43] Becker LC . Conditions for vasodilator-induced coronary steal in experimental myocardial ischemia. Circulation. (1978) 57:1103–10. doi: 10.1161/01.cir.57.6.1103. PMID: 416923

[44] Jirtle RL . Chemical modification of tumour blood flow. Int J Hyperthermia. (1988) 4:355–71. doi: 10.3109/02656738809016490. PMID: 3290350

[45] Wu H Exner AA Krupka TM Weinberg BD Haaga JR . Vasomodulation of tumor blood flow: effect on perfusion and thermal ablation size. Ann BioMed Eng. (2009) 37:552–64. doi: 10.1007/s10439-008-9605-x. PMID: 19085107 PMC3294296

[46] Chakhoyan A Corroyer-Dulmont A Leblond MM Gérault A Toutain J Chazaviel L . Carbogen-induced increases in tumor oxygenation depend on the vascular status of the tumor: a multiparametric MRI study in two rat glioblastoma models. J Cereb Blood Flow Metab. (2017) 37:2270–82. doi: 10.1177/0271678X16663947. PMID: 27496553 PMC5464716

[47] Bocca P Di Carlo E Caruana I Emionite L Cilli M De Angelis B . Bevacizumab-mediated tumor vasculature remodelling improves tumor infiltration and antitumor efficacy of GD2-CAR T cells in a human neuroblastoma preclinical model. Oncoimmunology. (2017) 7:e1378843. doi: 10.1080/2162402X.2017.1378843. PMID: 29296542 PMC5739560

[48] Ma W Wang Y Zhang R Yang F Zhang D Huang M . Targeting PAK4 to reprogram the vascular microenvironment and improve CAR-T immunotherapy for glioblastoma. Nat Cancer. (2021) 2:83. doi: 10.1038/s43018-020-00147-8. PMID: 35121889 PMC10097424

[49] Zhou M Chen M Shi B Di S Sun R Jiang H . Radiation enhances the efficacy of EGFR-targeted CAR-T cells against triple-negative breast cancer by activating NF-κB/Icam1 signaling. Mol Ther. (2022) 30:3379–93. doi: 10.1016/j.ymthe.2022.07.021. PMID: 35927951 PMC9637637

[50] DeSelm C Palomba ML Yahalom J Hamieh M Eyquem J Rajasekhar VK . Low-dose radiation conditioning enables CAR T cells to mitigate antigen escape. Mol Ther. (2018) 26:2542–52. doi: 10.1016/j.ymthe.2018.09.008. PMID: 30415658 PMC6225039

[51] Weiss T Weller M Guckenberger M Sentman CL Roth P . NKG2D-based CAR T cells and radiotherapy exert synergistic efficacy in glioblastoma. Cancer Res. (2018) 78:1031–43. doi: 10.1158/0008-5472.CAN-17-1788. PMID: 29222400

[52] Wang T Zhang K You F Ma R Yang N Tian S . Preconditioning of radiotherapy enhances efficacy of B7-H3-CAR-T in treating solid tumor models. Life Sci. (2023) 331:122024. doi: 10.1016/j.lfs.2023.122024. PMID: 37574043

[53] Kostopoulos N Costabile F Krimitza E Beghi S Goia D Perales-Linares R . Local radiation enhances systemic CAR T-cell efficacy by augmenting antigen crosspresentation and T-cell infiltration. Blood Adv. (2024) 8:6308–20. doi: 10.1182/bloodadvances.2024012599. PMID: 39213422 PMC11700247

[54] Puebla-Osorio N Fowlkes NW Barsoumian HB Xega K Srivastava G Kettlun-Leyton C . Enhanced tumor control and survival in preclinical models with adoptive cell therapy preceded by low-dose radiotherapy. Front Oncol. (2024) 14:1407143. doi: 10.3389/fonc.2024.1407143. PMID: 39445067 PMC11496962

[55] Quach HT Skovgard MS Villena-Vargas J Bellis RY Chintala NK Amador-Molina A . Tumor-targeted nonablative radiation promotes solid tumor CAR T-cell therapy efficacy. Cancer Immunol Res. (2023) 11:1314–31. doi: 10.1158/2326-6066.CIR-22-0840. PMID: 37540803 PMC10592183

[56] Zhao X Yang J Zhang X Lu XA Xiong M Zhang J . Efficacy and safety of CD28- or 4-1BB-based CD19 CAR-T cells in B cell acute lymphoblastic leukemia. Mol Ther Oncolytics. (2020) 18:272–81. doi: 10.1016/j.omto.2020.06.016. PMID: 32728615 PMC7378699

[57] Cappell KM Kochenderfer JN . A comparison of chimeric antigen receptors containing CD28 versus 4-1BB costimulatory domains. Nat Rev Clin Oncol. (2021) 18:715–27. doi: 10.1038/s41571-021-00530-z. PMID: 34230645

[58] Ying Z He T Wang X Zheng W Lin N Tu M . Parallel comparison of 4-1BB or CD28 co-stimulated CD19-targeted CAR-T cells for B cell non-Hodgkin’s lymphoma. Mol Ther Oncolytics. (2019) 15:60–8. doi: 10.1016/j.omto.2019.08.002. PMID: 31650026 PMC6804784

[59] Amatya C Pegues MA Lam N Vanasse D Geldres C Choi S . Development of CAR T cells expressing a suicide gene plus a chimeric antigen receptor targeting signaling lymphocytic-activation molecule F7. Mol Ther. (2021) 29:702–17. doi: 10.1016/j.ymthe.2020.10.008. PMID: 33129371 PMC7854354

[60] Textor A Grunewald L Anders K Klaus A Schwiebert S Winkler A . CD28 co-stimulus achieves superior CAR T cell effector function against solid tumors than 4-1BB co-stimulus. Cancers (Basel). (2021) 13:1050. doi: 10.3390/cancers13051050. PMID: 33801448 PMC7958604

