# Supplementary material for: Ionizing radiation enhances CAR T-cell infiltration and efficacy in solid tumors
Source: Front Immunol. 2026 Apr 15;17:1704419. doi: 10.3389/fimmu.2026.1704419 (PMC13125132; doi:10.3389/fimmu.2026.1704419)
Supplement: Supplementary file 1 [file DataSheet1.pdf]

Supplementary Figure 1

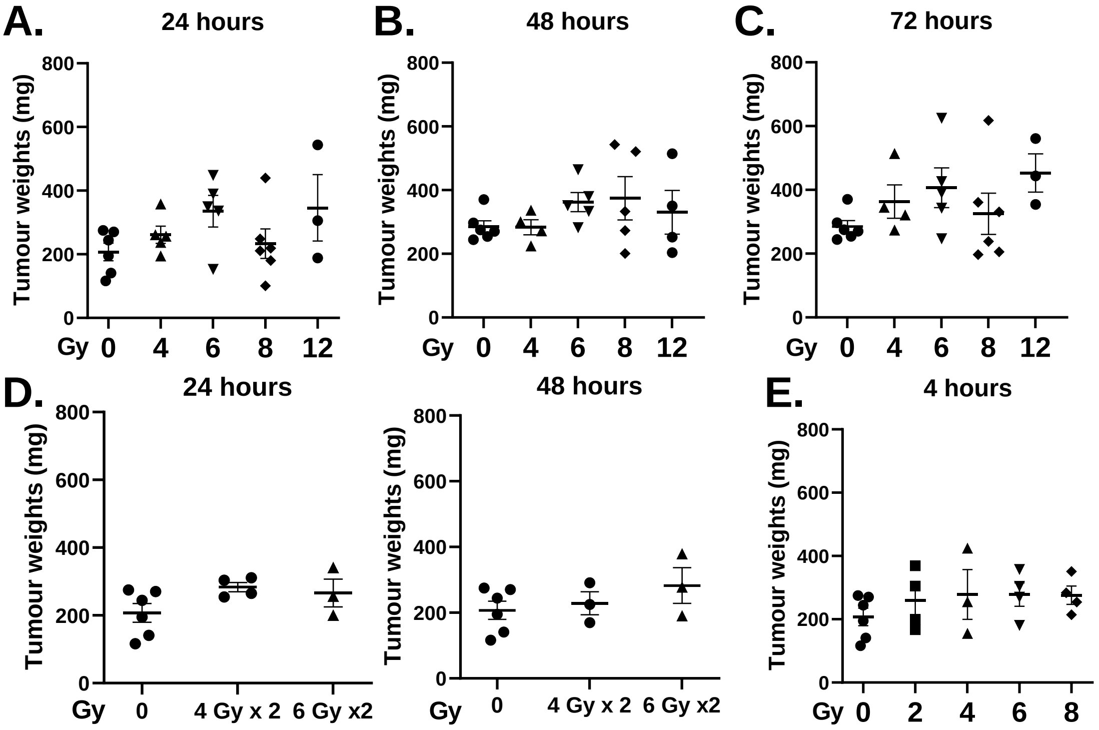

Supplementary Figure 1. (A-C) Tumour weights at time of cocktail dye delivery and tumour resection following single dose irradiation. Data points are individual tumours with n = 3-5 mice (IR groups) to 6 mice (no-IR group) per group. **(D)** Tumour weights at time of cocktail dye delivery and tumour resection following double dose irradiation. Data points are individual tumours with n = 3-5 mice (IR groups) to 6 mice (no-IR group) per group. **(E)** Tumour weights at time of cocktail dye delivery and tumour resection 4 hours post single irradiation doses. Data points are individual tumours with n = 4-5 mice (IR groups) to 6 mice (no-IR group) per group.

Supplementary Figure 2

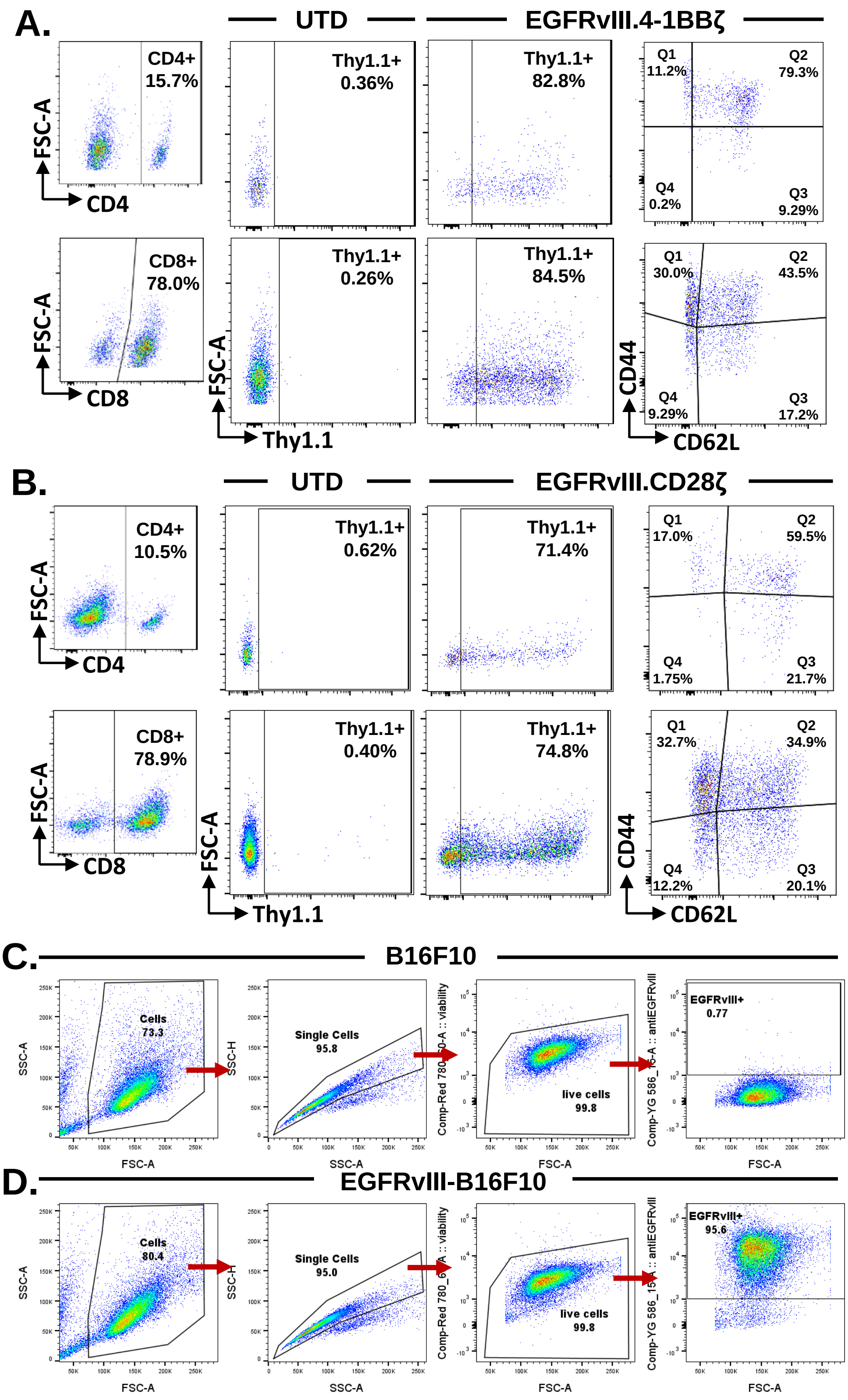

Supplementary Figure 2. (A) Representative flow cytometry analysis of EGFRvIII.4-1BBζ CAR T cell transduction. Thy1.1, CD44, and CD62L expression in CD4 T cells (top) and CD8 T cells (bottom) 24 hours after retroviral transduction. **(B)** Representative flow cytometry analysis of EGFRvIII.CD28ζ CAR T cell transduction. Thy1.1, CD44, and CD62L expression in CD4 T cells (top) and CD8 T cells (bottom) 24 hours after retroviral transduction. **(C-D)** Confirmation of CAR target on tumour cells on (C) wildtype cells and (D) on EGFRvIII-B16F10 cells.

Supplementary Figure 3

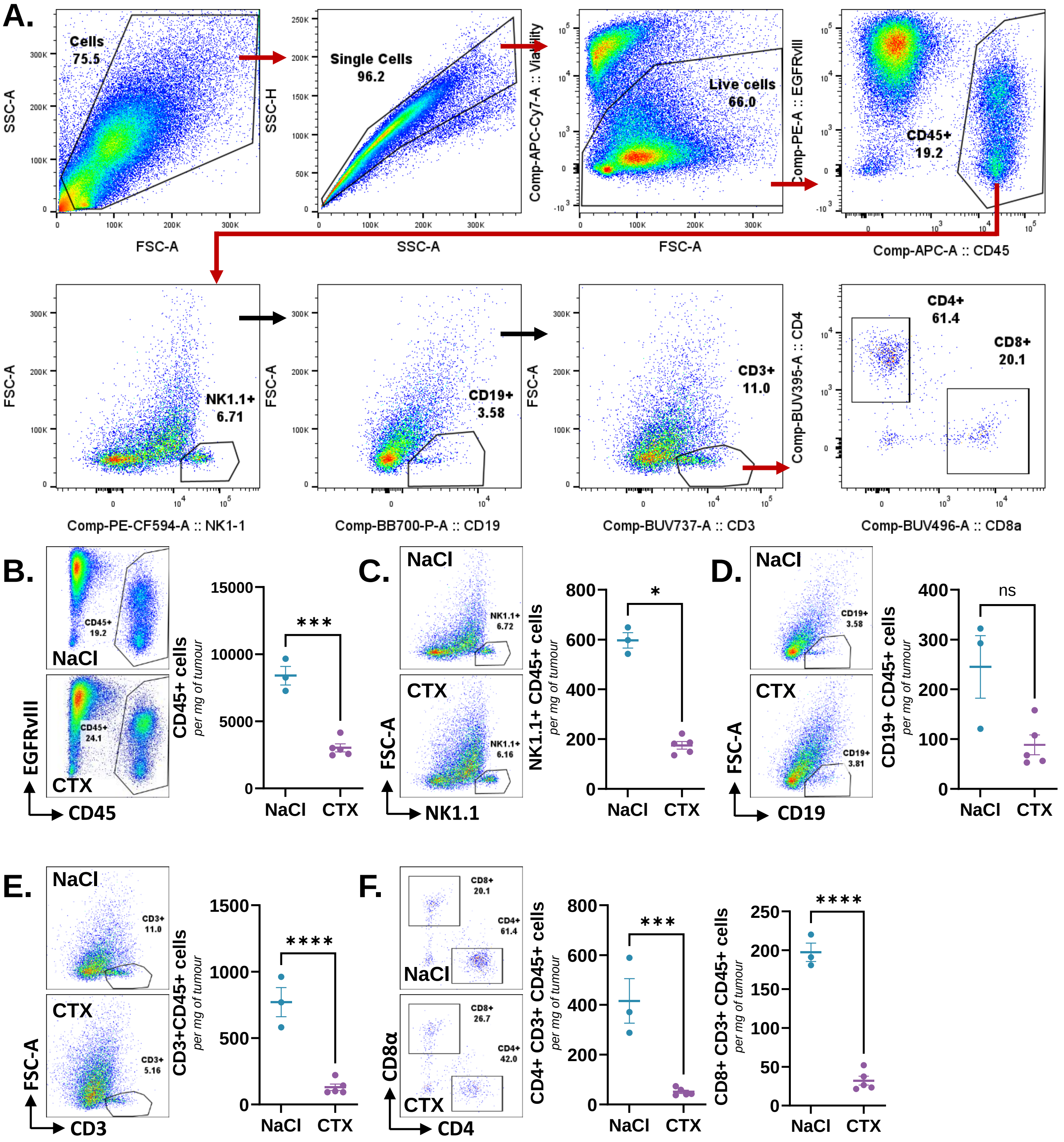

Supplementary Figure 3. (A) Representative flow plots showing gating strategy to analyze lymphoid cell populations from processed EGFRvIII-B16F10 tumour samples lymphodepleted three days prior with 200 mg/kg of CTX. **(B)** Number of leukocytes per mg of tumour and representative flow plots. **(C)** Number of NK cells per mg of tumour and representative flow plots. **(D)** Number of B cells per mg of tumour and representative flow plots. **(E)** Number of CD3+ cells per mg of tumour and representative flow plots. **(F)** Number of CD4+ (left) and CD8+ (right) cells per mg of tumour and representative flow plots. Data points are individual mice with mean ± SEM of 3-5 mice/group. *$p \leq 0.05$; ***$p \leq 0.001$; ****$p \leq 0.0001$.

Supplementary Figure 4

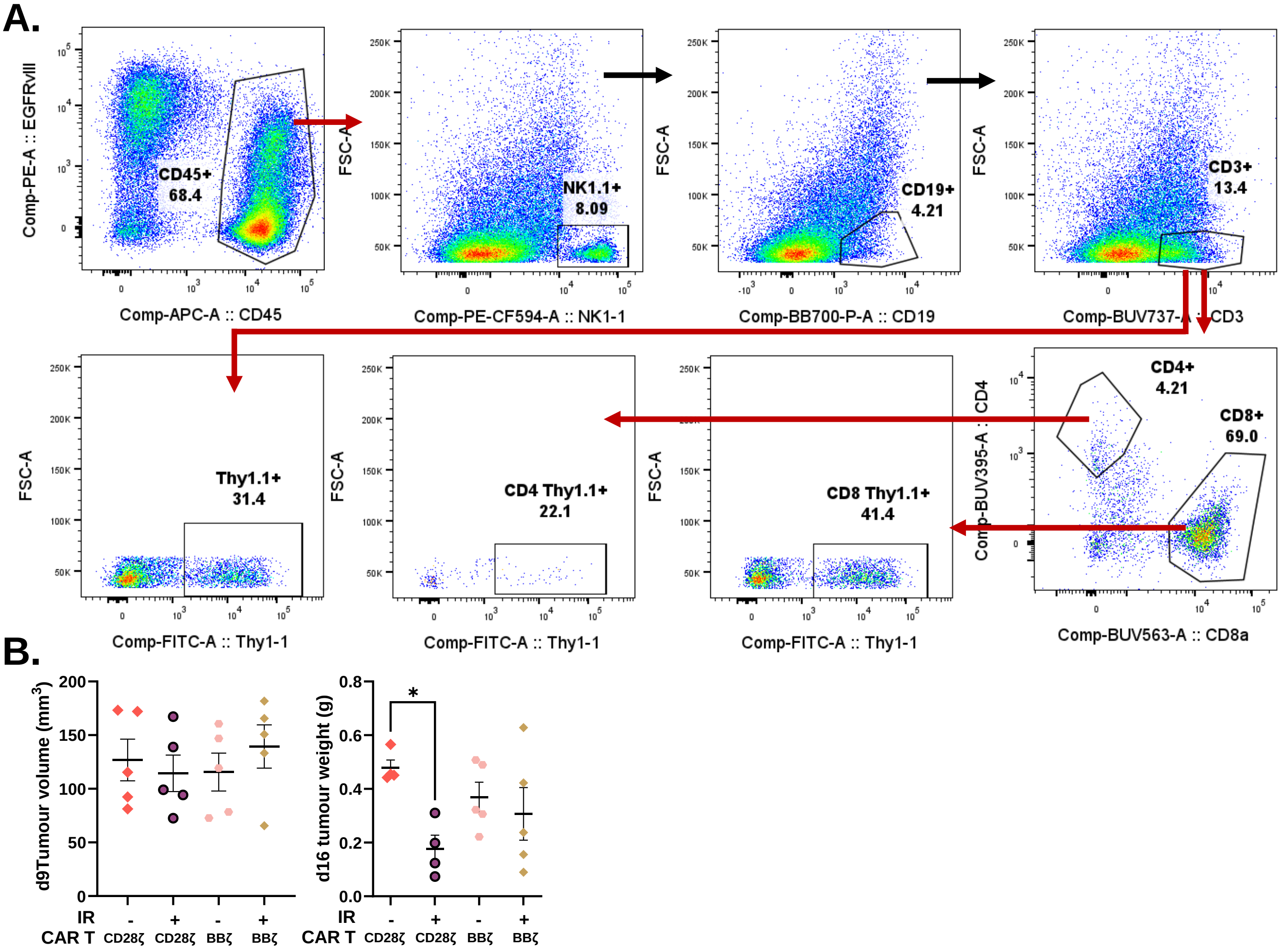

Supplementary Figure 4. (A) Representative flow plots showing gating strategy to analyze tumour-infiltrating BBζ and CD28ζ CAR T cells. **(B)** Tumour volume on day 9 (left) and tumour weight at on day 16 post-implant (right) of *in vivo* experiment looking at longer-term accumulation of 1.0 x 10^6 CD28ζ CAR T and 10.0 x 10^6 BBζ CAR T with and without 8 Gy. N= 5 mice/group.

Supplementary Figure 5

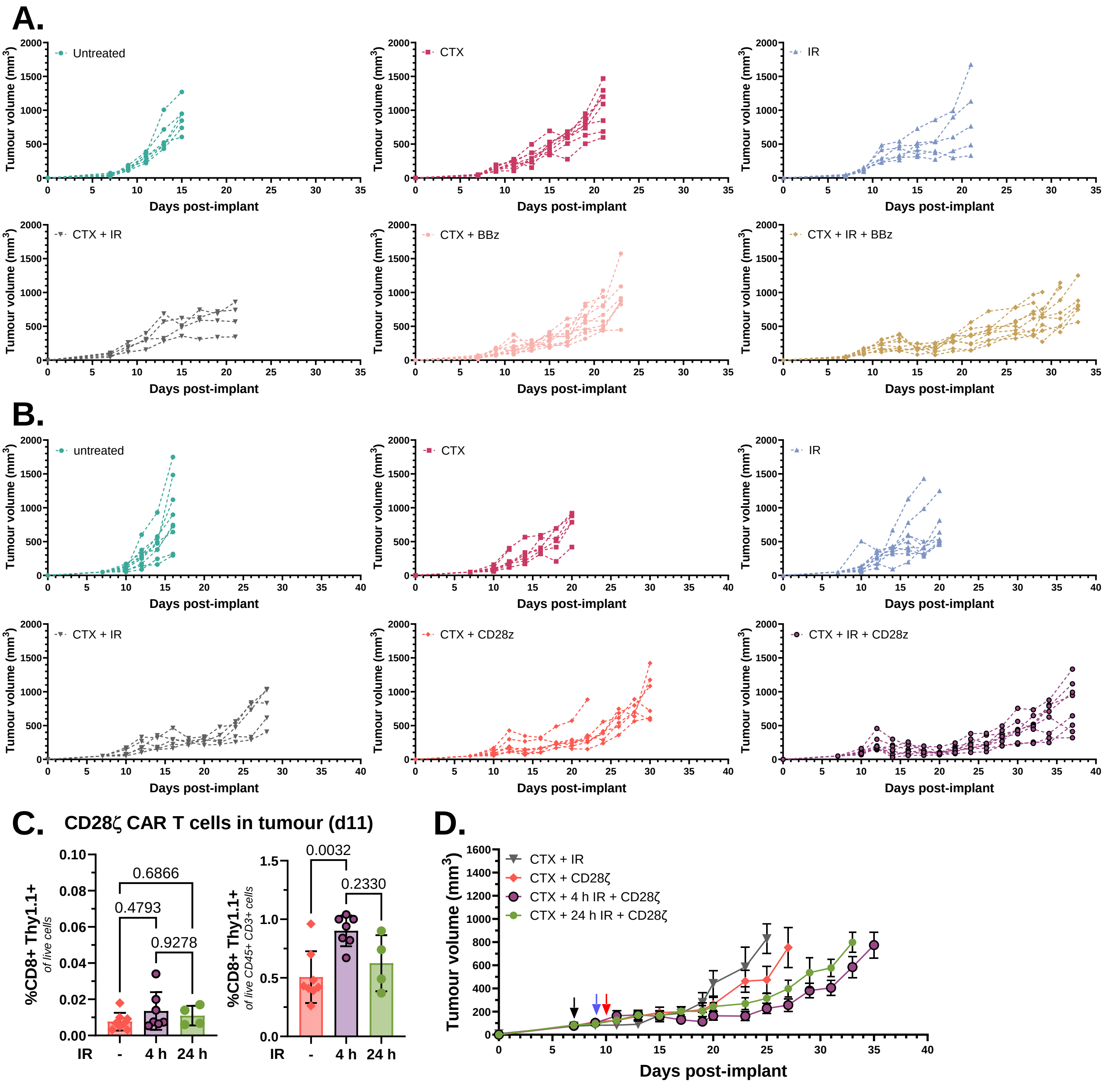

Supplementary Figure 5. (A) Individual tumour growth curves of all the treatment groups for *in vivo* experiment, CTX = 200 mg/kg cyclophosphamide, IR = 8 Gy, BBζ = 10.0 x 10^6 EGFRvIII.4-1BBζ CAR T cells. N = 5 -11 mice/group. **(B)** Individual tumour growth curves of all the treatment groups for *in vivo* experiment, CTX = 200 mg/kg cyclophosphamide, IR = 8 Gy, CD28ζ = 1.0 x 10^6 EGFRvIII.CD28ζ CAR T cells. N= 5-9 mice/group. **(C)** Comparing presence of tumour-infiltrating CD28ζ CAR T cells 24 hours after initial delivery between tumours that received a single 8 Gy 4-hours or 24-hours prior to CAR T cells. N= 4 – 6 mice/group. **(D)** Tumour growth delay curves comparing 4-hour interval and 24-hour interval between 8 Gy and CAR T cell delivery (1.0 x 10^6 CD28ζ). Data points are mean ± SEM of 5-8 mice/group.
